# Neurorehabilitation-Based Movement Representation Techniques in the Management of Craniocervical and Orofacial Pain: A Systematic Review of Randomized Controlled Trials

**DOI:** 10.3390/life16010145

**Published:** 2026-01-15

**Authors:** Alberto García-Alonso, Luis Polo-Ferrero, Ana Silvia Puente-González, Tamara Manso-Hierro, Marta Beatriz Carrera-Villegas, Roberto Méndez-Sánchez

**Affiliations:** 1 Department of Nursing and Physiotherapy, University of Salamanca, 37007 Salamanca, Spain; albertogarciaalonso@usal.es (A.G.-A.); silviapugo@usal.es (A.S.P.-G.); tamara.manso@usal.es (T.M.-H.); martacv28@usal.es (M.B.C.-V.); ro_mendez@usal.es (R.M.-S.); 2 Institute of Biomedical Research of Salamanca (IBSAL), 37007 Salamanca, Spain

**Keywords:** orofacial, craniomandibular, pain, temporomandibular, motor imagery, action observation

## Abstract

Background: Craniocervical pain and temporomandibular disorders (TMDs) are prevalent, interconnected conditions. While Movement Representation Techniques (MRTs) are cognitive interventions targeting central pain mechanisms, their specific efficacy here lacks synthesis. This study systematically analyzes the effectiveness of MRTs, such as motor imagery (MI) and action observation (AO), on pain and function in individuals with craniocervical and orofacial pain. Methods: A systematic review of RCTs (PROSPERO: CRD420251155428) was conducted following PRISMA guidelines. Four databases were searched for studies applying MRTs (MI, AO, laterality discrimination) to adults with craniocervical or orofacial pain. Primary outcomes were pain and functionality. Methodological quality was assessed using the PEDro scale and Cochrane RoB 2 tool. Results: Eight RCTs (*n* = 362) were included. Methodological quality was high (PEDro scores 8–9). MRTs significantly increased Pressure Pain Threshold (PPT) in the masseter, trapezius, and cervical regions. Functional improvements included enhanced cervical range of motion and sensorimotor control. AO consistently demonstrated superior outcomes. However, results for orofacial variables were derived from asymptomatic subjects. Results for cervical muscle strength were inconsistent. Conclusions: MRTs, especially AO, show potential to reduce pain and improve function in craniocervical disorders. Evidence in symptomatic orofacial pain populations is non-existent. Protocol heterogeneity and limited research groups necessitate further high-quality, multicenter RCTs to establish robust clinical guidelines.

## 1. Introduction

Temporomandibular disorders (TMDs) are prevalent musculoskeletal conditions affecting about 34% of the population, and this is projected to reach 44% [1]. Closely linked to cervical pain and oral parafunctions [2], TMDs are considered one of the most common causes of musculoskeletal orofacial pain [3,4], with a three- to fivefold higher prevalence in women [5]. Beyond their frequency, they markedly impair quality of life and entail substantial socioeconomic costs [5]. Psychological factors such as stress, anxiety, poor sleep, along with physical factors like physical inactivity, obesity, and low muscle strength—often accompanied by fear of movement—contribute to their chronicity [2,5,6].

Conservative management remains the first-line approach, focusing on pain reduction and functional improvement through manual therapy, exercise, behavioral strategies, acupuncture, or laser therapy [3,7]. However, many of these interventions lack robust evidence, particularly for chronic orofacial pain [8,9,10].

Recently, approaches targeting central pain mechanisms—such as pain neuroscience education and motor imagery (MI)—have gained attention [8]. The broader term Movement Representation Techniques (MRTs) encompasses MI, action observation (AO), and visual feedback therapy (VFT), which induce cognitive representations of movement and activate cortical networks similar to motor execution. These non-invasive methods have shown benefits in neurological and musculoskeletal conditions, enhancing range of motion and strength [11]. It is important to note that most evidence for MI efficacy comes from neurological rehabilitation [12], and its specific application in TMD remains less explored.

MI involves mental simulation of movement to improve motor learning [13], while AO leverages the mirror neuron system through observation of specific movements to promote cortical reorganization [14]. VFT, using mirrors, videos, or virtual reality, provides visual feedback facilitating visuoproprioceptive integration [11].

Despite promising mechanisms, the use of MRTs in cranio-cervical and orofacial regions remains limited. Most studies have focused on the upper limbs or neuropathic pain, and evidence in TMDs or cervical pain is scarce [8,13]. The close anatomical and functional relationship between the temporomandibular joint (TMJ) and cervical region supports the rationale for combined interventions. The TMJ, as part of the stomatognathic system, can generate referred pain and cervical movement limitations [15,16,17], mediated by the trigeminocervical nucleus, where nociceptive afferents from the trigeminal nerve converge with spinal afferents from C1–C3, creating overlapping receptive fields that facilitate referred pain between the head and neck [18,19]. This interconnection supports integrated rather than isolated therapeutic approaches.

However, existing studies show methodological limitations, including small samples, heterogeneous protocols, and unclear mechanisms. Given the shared neurophysiological basis of TMDs and cervical pain, synthesizing current evidence is essential. This systematic review aims to summarize and critically analyze the effectiveness of MRTs in orofacial and cranio-cervical pain, outlining their clinical relevance and research gaps in this emerging field.

## 2. Materials and Methods

### 2.1. Protocol and Registration

This systematic review of randomized controlled trials was conducted following the guidelines recommended by the Preferred Reporting Items for Systematic Reviews and Meta-Analyses (PRISMA) Statement [20]. The review protocol was registered in the International Prospective Register of Systematic Reviews (PROSPERO) to ensure methodological transparency and avoid duplication (registration ID: CRD420251155428).

### 2.2. Eligibility Criteria

The PICOS strategy (Population, Intervention, Comparison, Outcomes, and Study design) [21] was followed to establish the eligibility criteria. The review established the year of publication of the studies as a criterion, considering only those between 2018 and 2024. Studies conducted in people with acquired brain damage or spinal cord injury and studies that do not use pain variables or craniocervical or craniomandibular function as outcome variables were excluded.

‐ *Population:* Subjects who have performed MRTs with an age between 18 and 65 years.
‐ *Intervention:* Eligible interventions were any MRT (MI, laterality discrimination, AO, visual feedback) applied to the craniomandibular region and/or the craniocervical region.
‐ *Comparison:* No intervention, placebo, or conventional interventions for pain and dysfunction in the orofacial and cervical region.
‐ *Outcomes:* Reduction in pain and improvement in functionality (joint range, muscle strength, etc.).
‐ *Study design:* Only randomized clinical trials (RCTs) will be considered for inclusion.

### 2.3. Search Strategy

A comprehensive literature search was conducted from 1 to 15 April 2024 across four databases: PubMed, Scopus, Web of Science (WOS) and EBSCO Host. Different descriptors and keywords were considered using the Boolean operators ‘AND’ and ‘OR’ to structure the search and refine the results. The complete search query applied in each database is available in Supplementary Data S1.

### 2.4. Study Selection

Study selection was conducted by two reviewers (AGA and RMS) reading the articles independently. Both researchers thoroughly reviewed the publications evaluated for eligibility, rigorously considering the aforementioned eligibility criteria. Subsequently, a comparison of the independent evaluations was carried out, and both researchers fully agreed on the inclusion and exclusion decisions, so it was not necessary for a third reviewer to intervene in the final inclusion decision (a third evaluator (LPF) was already planned).

### 2.5. Data Extraction

Data extraction from the selected studies was carried out by one researcher, using a standardized form. The following information was considered in each study: authors, year of publication, type pf study, participants, total *n*, age, sex, intervention group (IG), control group (CG), variables to be measured, assessment tools, measurements, duration of the intervention, and results. The collection and analysis of data from the eight selected articles is presented in Table 1.

### 2.6. Data Synthesis and Analysis

Due to the heterogeneity of the included studies regarding the variability in intervention dosages (ranging from single-session immediate effects to 4-week training programs), the diversity of measurement tools used for the same outcome (e.g., different algometers and pain scales), and inconsistent follow-up periods, a quantitative synthesis (meta-analysis) was not feasible. Therefore, a descriptive synthesis was performed. The results were grouped and presented narratively according to the primary outcomes (pain and function) and the type of intervention. Risk of bias due to missing results (publication bias) was not assessed via funnel plots primarily due to the limited number of included studies, following standard recommendations.

### 2.7. Risk of Bias and the Assessment of Methodological Quality of the Studies

The PEDro scale was used to assess the quality of the evidence. This scale scores are according to the presence of items of evidence quality (1 point) or the absence of those items (0 points), up to a total score of 10 points [30]. This total score is achieved by adding the scores for items 2 to 11. The 11 items assess eligibility criteria and external validity (item 1), internal validity (items 2–9) and statistical reporting (items 10 and 11). A score below 4 is considered poor quality, between 4 and 5 is fair, 6 to 8 is good, and 9–10 is excellent.

The risk of bias was additionally evaluated using version 2 of the Cochrane Risk of Bias tool for randomized trials (RoB 2) [31]. Each study was assessed according to the predefined criteria of this instrument, which encompasses six domains: the randomization process, deviations from intended interventions, missing outcome data, measurement of outcomes, selection of the reported results, and overall risk of bias.

## 3. Results

### 3.1. Search Results and Study Selection

Following the search strategy and applying the selection criteria, a total of 492 articles were obtained: 91 in PubMed, 115 in Scopus, 174 in Web of Science (WOS), and 117 in EBSCOhost.

**Figure 1:**
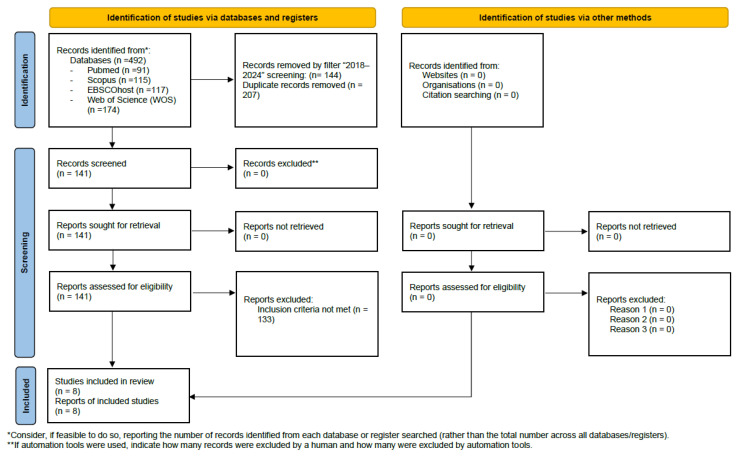
Prisma 2020 Flow Diagram.

The filter ‘2018–2024’ was applied to each database, yielding a total of 348 articles: 65 in PubMed, 82 in Scopus, 115 in WOS, and 86 in EBSCOhost. 207 of the articles were duplicates, so 141 articles were selected. The Mendeley Reference Manager bibliographic management software (v 2.141.0, Elsevier, Amsterdam, The Netherlands) was used to discard duplicate articles. Finally, a total of 8 studies were included in the systematic review. The process of study identification, screening, and inclusion is summarized in the PRISMA flow diagram (Figure 1).

### 3.2. Study Characteristics

#### 3.2.1. Sample Size and Population Description

The total number of subjects included in the eight selected articles is 362, with sample sizes ranging from 30 subjects in two articles [23,29], to 60 subjects in another study [27]. In all the studies reviewed, the sample consisted of individuals aged between 18 and 65, with the exception of one [22], where the participants were university students aged between 18 and 22. In terms of gender distribution within the studies analyzed, there was a predominance of female participants in most articles, except for one study, where there was a higher proportion of men than women [25]. Overall, there is a higher proportion of women, at 59%, compared to 41% of men.

#### 3.2.2. Clinical Profile and Inclusion Criteria

Three of the eight articles were conducted on asymptomatic subjects [25,26,28]. The other articles were conducted on subjects with neck pain. All of them had been diagnosed by a family doctor or specialist, except in the study by Al Shrbaji et al., which included patients with chronic idiopathic neck pain defined as recurrent or persistent pain lasting more than 3 months, without trauma or associated etiology or diagnosis, arising anywhere between the upper nuchal line and an imaginary transverse line passing through the first thoracic spinous process and the lateral edges of the neck [27]. One study also included subjects diagnosed with cervical pain lasting at least 3 months [22]. However, 3 studies included subjects diagnosed with cervical pain lasting at least 6 months [23,24,29]. It should be noted that most studies excluded patients with systemic, rheumatic, cardiorespiratory, and neurological diseases. Subjects who had undergone surgery in the cervical area were also excluded.

#### 3.2.3. Study Duration and Frequency of Intervention

The duration of the studies was consistent in most cases, although it is important to note that it ranged from 4 weeks in one article [22] to a single day in 4 of the studies considered [23,24,27,29].

The frequency of interventions ranged from a single session in the four articles cited above to five weekly sessions in the study by Özcan et al. [22]. The duration of the interventions varied widely, from 60 min [22], to 4 min [23,29], as well as in one study, where participants viewed 20 images, each for only 4 s [24].

#### 3.2.4. Group Allocation and Experimental Design

Subjects were randomly assigned to different groups. The distribution varied between studies: 50% of the articles opted for a division into two groups [22,24,26,27], while the other 50% opted for a distribution into three groups [23,25,28,29].

#### 3.2.5. Description of Control and Intervention Groups

Most studies used exercise programs or physical activity sessions as a CG. However, some studies opted to use observation of videos showing natural landscapes as an alternative to compare with observation of specific actions. In the IGs, researchers mainly used MI, AO, and laterality discrimination techniques.

#### 3.2.6. Outcome Variables and Measurement Instruments

Of the eight articles selected for the study, a total of 26 variables were examined and evaluated. These variables covered different aspects, providing a wide range of data, and are listed in Table 1. The articles analyze between twelve [25], and four variables [22]. The 26 variables identified across the studies assess a broad spectrum of domains, which can be classified into several main groups: pain-related variables (e.g., pressure pain threshold, pain intensity); range of motion, muscle activation, and functional variables (e.g., active cervical ROM, neck muscle strength); motor imagery and sensorimotor control variables (e.g., ability to generate mental images, sense of neck repositioning); psychosocial variables (e.g., fear of movement, pain catastrophising); and other key variables (e.g., neck disability, level of physical activity, and quality of life).

Different measurement tools were used to analyze these 26 variables assessed in the included studies. These variables can be grouped as follows:

‐ Pain-related variables: Pressure Pain Threshold (PPT) in different muscles or areas, measured with a mechanical pressure algometer [23,25,26,27,28]; pain intensity, assessed using the Visual Analogue Scale (VAS) [22,23,24,27] and conditioned pain modulation, evaluated with an algometer and occlusion cuff [25].
‐ Range of motion, muscle activation, and functional variables: Active cervical ROM (Range of Motion) assessed with a CROM goniometer [24,25]; neck muscle strength and endurance evaluated with a hand dynamometer (Advanced Force Gauge) [27] and isometric test [25]; tongue muscle strength measured using the Iowa Oral Performance Instrument (IOPI) [26,28]; tongue length with a ruler and depressor [28]; maximum mouth opening with the craniomandibular scale [28];
‐ Motor imagery and sensorimotor control variables: Ability to generate mental motor images evaluated with the Revised Movement Imagery Questionnaire (MIQ-R) [23,25,26,28,29]; timing of imagined movements or mental timing with a stopwatch [23,28,29]; sense of neck repositioning measured with the Sensory Motor Control Oriented Rehabilitation IAOM-US [29] or the Motion Guidance Clinic Kit for JPE [24]; ability to perform laterality discrimination tasks assessed using the Recognise Online application [24]; influence of imagination evaluated with the Movement Imagery Questionnaire-3 (MIQ-3) [22].
‐ Psychosocial variables: Fear of movement related to pain, assessed with the Tampa Scale for Kinesiophobia [23,24,25,27,29]; fear avoidance beliefs, with the Fear Avoidance Beliefs Questionnaire (FABQ) [25,27]; degree of pain catastrophising, with the Pain Catastrophising Scale [23,24,25,27]; anxiety, with the State-Trait Anxiety Inventory, (STAI) [25]; depression, with the Beck Depression Inventory-II (BDI-II) [25]; attention and cognitive function evaluated with the modified Stroop test with Encephalapp [25].
‐ Other variables: neck disability, using the Neck Disability Index [22,23,24,27,29]; level of physical activity assessed with the International Physical Activity Questionnaire (IPAQ) [23,25,26,28,29]; tactile acuity measured with an aesthesiometer (Baseline Two Point Aesthesiometer) [27]; perceived exertion measured using the Borg scale [26]; quality of life using the Short Form 36 Health Survey (SF-36) [22]; heart rate recorded with a Garmin Forerunner VR 225 [23] and the ability to engage in regular physical activity, assessed with the Physical Activity Self-Efficacy Scale [28].

The variables were measured before and after the intervention in all studies. In addition, in two studies, an additional measurement was taken 10 min after the intervention to assess immediate effects and possible short-term changes [23,29]. On the other hand, other authors implemented two interventions in their study, which involved taking two pre-intervention measurements and two post-intervention measurements for each session [25]. In the study by La Touche et al., three post-intervention measurements were taken, one after each session, in addition to the initial measurement taken before the intervention [28].

### 3.3. Summary of Results

#### 3.3.1. Effects on Pain

The results showed an improvement in pain, evidenced by an increase in PPT in the masseter, trapezius, and cervical regions [23,25,28]. Another study also reported significant differences in PPT, with the group performing moderate training showing higher PPT values than the comparison group [26]. Morales et al. found no significant differences in distant pain modulation, as no changes in PPT were observed in the tibial region [25]. Conversely, another study reported that action observation led to greater pain modulation both locally and remotely [23]. It should be noted that two studies reported decreased pain values in both groups after the intervention [22,27], although one of them found no significant differences in PPT [27].

#### 3.3.2. Effects on Cervical Function

Several studies documented improvements in cervical function. Specially, cervical ROM were enhanced in groups performing laterality discrimination with neck images [24] and cervical exercises with craniocervical flexion [25]. Furthermore, a greater increase in left rotation movement was also found in the MI and AO groups compared to the placebo observation group [29]. Statistically significant improvements in cervical joint repositioning were also reported in two separate studies [24,29].

#### 3.3.3. Motor Imagery Ability

Evidence from three studies indicates that interventions can enhance motor imagery ability [22,25,28]. For instance, both exercise group and MI plus exercise group demonstrated increased motor visualization ability, along with kinesthetic and internal visual images [22]. In the AO group, significant changes were found in the ability to generate motor images [25], and both the AO and MI groups, along with the CG, improved on the visual subscale of the MIQ-R. In addition, only the AO group showed improvements in the timing of mental images [28].

#### 3.3.4. Muscle Strength

Findings regarding muscle strength were inconsistent or highly specific. In terms of tongue muscle strength, one study found that only AO produced changes [28]. Significant differences were found only within the CG between measurements before and after the first session [26]. On the other hand, other authors claim that AO had no effect on the muscle strength of the cervical musculature [27].

#### 3.3.5. Heart Rate

One study identified differences between the AO and MI groups in HR response. Notably, the AO intervention appeared to elicit a greater cardiac response, increasing HR compared to MI [23].

### 3.4. Methodological Quality

The methodological rigor of the included studies was assessed using the PEDro scale. Scores across the eight studies ranged from 8 to 9, reflecting generally high quality. Specifically, two studies achieved a score of 9 points [25,28], while the remaining six studies scored 8 points [22,23,24,26,27,29].

Most trials exhibited strengths in core design elements, including random allocation, comparable baseline characteristics, and blinded outcome assessment, which support the internal validity of their findings. However, all studies lacked blinding of participants, a limitation frequently encountered in trials of non-pharmacological interventions. In addition, some reports provided limited detail regarding allocation concealment, potentially introducing a small risk of bias. A complete overview of PEDro scores for each study is shown in Table 2.

### 3.5. Risk of Bias

**Figure 2:**
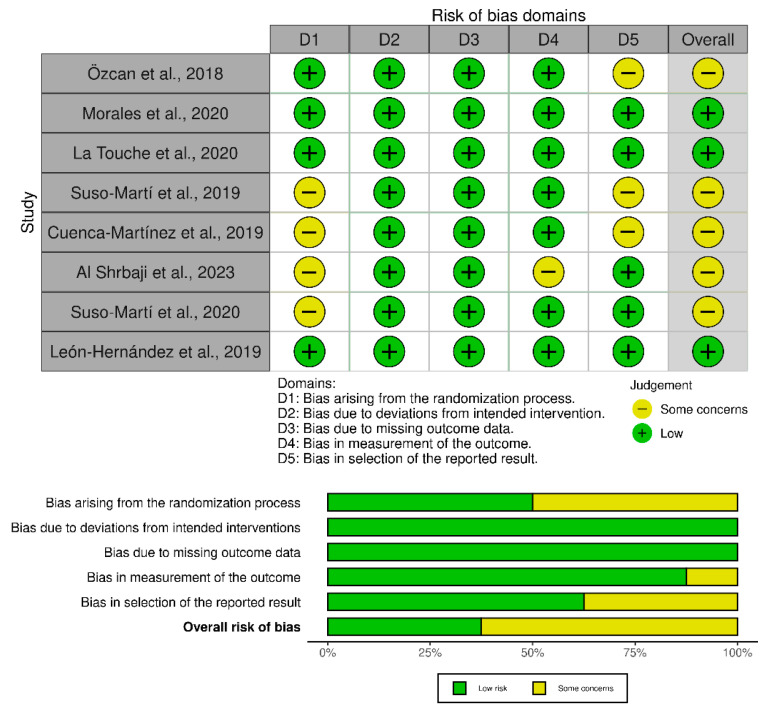
Risk of bias assessment for the included studies [22,23,24,25,26,27,28,29].

The risk of bias assessment was performed using the Cochrane Rob 2 tool for randomized parallel trials for all studies. Three studies were judged low risk across all domains [24,25,28], while the remaining five were rated overall as “some concerns” [22,23,26,27,29]. The most frequent limitations affected D1 (randomization process) and D5 (selection of the reported result. Minor issues in D3 isolated concerns in D3 (incomplete outcome data) were also noted. Importantly, none of the included studies were rated as having a high risk of bias. Overall, these findings suggest a generally sound methodological quality, and while some reporting aspects could be improved, they are unlikely to have materially affected the validity of the results. A summary of the overall assessments is presented in Figure 2.

## 4. Discussion

This review demonstrates that MRTs such as AO, MI, and laterality discrimination can reduce pain and improve function in individuals with craniocervical and orofacial pain. The main findings include increased PPT in the masseter, trapezius, and cervical regions, improvements in cervical ROM, and enhanced sensorimotor performance through better mental chronometry and motor imagery accuracy, particularly in AO and MI interventions.

Given the high prevalence and significant impact on the health and quality of life of patients with TMD [2,3,4,5,7], along with the chronicity and recurrence of neck pain [32], new therapeutic strategies are essential for managing cranio-cervical and orofacial pain. MRTs can improve the quality of life of patients with neck, headache, and orofacial pain [33]. The predominance of female participants (59%) in the included studies aligns with the higher prevalence of TMD and cervical disorders among women [5].

The improvements in pain sensitivity and ROM observed in most studies [23,24,26,28,29] suggest that MRTs may potentially modulate musculoskeletal and neurophysiological mechanisms involved in chronic pain. Since pain and movement restriction are hallmark symptoms of TMD and neck disorders, often associated with altered motor control [10], the sensorimotor improvements observed through enhanced MI and chronometry support the hypothesis that cognitive-motor strategies might help restore cortical representation and neuromotor control. However, it is important to note that most included studies did not employ direct neurophysiological assessment (e.g., EEG, fMRI or TMS); therefore, these mechanistic explanations should be interpreted as hypotheses rather than established evidence.

Two studies measured PPT at distant sites with differing results. Suso-Martí et al. measured PPT in the non-dominant lateral epicondyle and found that AO led to greater modulation both locally (trapezius and C2–C3 zygapophyseal joint) and remotely [23]. In contrast, Morales et al. found no significant differences in the tibial region, leaving uncertainty about the potential influence on central sensitization [25]. These conflicting results indicate that evidence regarding the remote hypoalgesic effects of MRTs is currently insufficient to draw firm conclusions.

However, statistical significance does not ensure clinical relevance. A minimum detectable change (MDC) of 47.2 kPa was established for the upper trapezius in patients with neck pain, suggesting that changes below this threshold may reflect measurement error rather than actual physiological improvement [34]. Furthermore, the predominance of single-session studies limits these findings to immediate and transient effects. Consequently, long-term clinical benefits remain unproven, highlighting the need for multi-session RCTs.

Differences were also observed regarding tongue and cervical muscle strength. AO produced no significant effects on cervical strength in the study by Al Shrbaji et al. [27], whereas La Touche et al. reported improvements in tongue strength exclusively in the AO group [28]. These findings suggest that the effectiveness of MRTs may depend on the specific muscle group or region affected by pain.

Variability was found in terms of the duration and frequency of interventions. While some studies conducted single sessions [23,24,27,29], others opted for programs lasting several weeks with longer sessions [22]. Despite these differences, results were consistently positive, suggesting that even short interventions may be beneficial. Six of the eight studies investigated the craniocervical region, where AO was consistently reported to produce superior outcomes [22,23,24,25,27,29], while the two studies addressing the craniomandibular region also demonstrated better outcomes for AO in tongue strength and chronometry [26,28].

It is essential to interpret these results with caution. Three of the included studies involved asymptomatic participants. Consequently, findings regarding orofacial outcomes in healthy subjects should not be directly extrapolated to patients with clinical TMD or chronic pain without further validation.

Although these techniques may serve as alternatives to exercise when pain is present, they appear more effective when combined with movement or simple exercises (MRTs). Further research is needed in symptomatic populations with larger samples and both short- and long-term follow-ups. Despite limited evidence, current studies support that AO increases corticospinal excitability in healthy adults, especially when combined with imitation and physical practice, improving observed motor performance [35].

Similarly, MI shares neural networks with actual movement execution, activating cortical areas involved in motor planning and programming. This explains why combining MI with physical exercise in patients with spinal cord injury has shown superior results compared with isolated interventions. However, recent studies have provided limited evidence regarding its effectiveness in upper-limb functional training after stroke [36]. The activation of cortical regions influencing the central nervous system and promoting neural plasticity supports the use of these techniques in sports, neurological disorders, and chronic pain [37].

Hidalgo-Pérez et al. conducted a clinical trial in which the population consisted of asymptomatic subjects [37]. This study provides new evidence on the effects of cervical motor control exercises and MI on sensorimotor variables in the cervical region. The combination of both techniques improved craniocervical neuromotor control (measured by deep flexor activation) and reduced perceived post-exertional fatigue, whereas motor control exercises alone produced no significant changes.

Uritani et al. examined responses to motor imagery tasks in individuals with and without TMD, finding slower reaction times in those with the disorder, suggesting that this condition may impair motor imagery in the orofacial region [38]. Similarly, La Touche et al. showed that AO improved mental chronometry, supporting its potential to enhance cognitive-motor performance in TMD patients [28].

The reviewed studies present limitations, including variability in design, intervention duration, frequency, and outcome measures. Future research should standardize protocols and employ consistent assessment tools to enhance validity and comparability. Moreover, studies involving patients with orofacial pain are needed, as the two existing studies were conducted in asymptomatic samples [26,28]. Furthermore, the sample sizes in the included studies (ranging from 30 to 60 participants) imply a statistical power limitation [39]. Another limitation is that most articles originated from the same research group, potentially introducing bias and limiting generalizability. This ‘research cluster bias’ may inflate the consistency of the effects and potentially limits the external validity and reproducibility of the findings across different populations and clinical settings. Therefore, encouraging broader research collaboration is crucial to obtain more consistent and representative results. Finally, given that no meta-analysis could be performed and the synthesis was purely qualitative, a formal GRADE assessment—designed to evaluate certainty in pooled effect estimates—was not applicable. Therefore, while methodological quality was assessed using PEDro and RoB-2, the certainty of evidence was not formally rated.

MRTs represent a promising and non-invasive therapeutic option for the management of craniocervical and orofacial pain. However, long-term studies are required to confirm the durability of the observed effects and their impact on patients’ quality of life. The integration of advanced technologies, such as virtual reality and artificial intelligence, could open new opportunities to optimize treatment delivery, enhance patient adherence, and personalize therapeutic approaches.

Clinically, MRTs should be considered as an adjunct to therapeutic exercise and patient education, rather than a standalone treatment, particularly for patients with kinesiophobia.

This review provides the first comprehensive synthesis of MRTs applied to cranio-cervical and orofacial pain, supporting their promising clinical potential to modulate pain and improve neuromotor function. However, due to the methodological limitations identified, these findings should be interpreted with caution. Evidence for symptomatic orofacial pain populations is currently absent; therefore, conclusions regarding TMD treatment are preliminary. Despite current limitations, MRTs represent an innovative, neuroscience-based therapeutic avenue. Future research should prioritize standardized protocols and multicenter clinical trials to establish robust, evidence-based guidelines and to clarify their long-term effectiveness.

## 5. Conclusions

MRTs, particularly AO, demonstrate preliminary evidence of their potential to reduce pain and improve functional outcomes in the craniocervical region. However, regarding the orofacial region, it is critical to note that current evidence is largely derived from asymptomatic participants. Consequently, these findings cannot be directly extrapolated to clinical populations with TMD or chronic orofacial pain. Current findings are promising but require confirmation in higher-quality trials involving symptomatic subjects. Moreover, they may serve as valuable complementary strategies to be integrated with other physiotherapeutic approaches, especially physical exercise, in the management of these disorders. However, the heterogeneity observed among the included studies—in terms of the number of sessions, intervention duration, and outcome measures—limits the strength and generalizability of the present conclusions. Further high-quality research with standardized protocols and larger samples is needed to confirm these findings and establish evidence-based clinical recommendations.

## Figures and Tables

**Table 1:** Characteristics of the Included Studies.

Author, Year	Type of Study	Participants	Total *n*	Age (Years)	Sex (%)	IG	CG	Variables to Be Measured	Assessment Tool	Measurements	Duration of the Intervention	Results
Özcan, 2018 [22]	RCT	40 subjects (18–22 years) with nonspecific neck pain of at least 3 months	40. IG: 20 CG: 20	19.9 IG: 20.1 CG: 19.7	Women: IG 75% CG 90% Men: IG 25% CG 10%	20 subjects performed MI training for a maximum of 15 min, 5 days a week, for 4 weeks after completing the exercise program, which the control group also completed. An MI component was implemented each week.	20 subjects performed cervical exercises (45 min/day, 5 days/week, 4 weeks). Exercises: craniocervical flexion/extension and isometric with elastic band (progressing from sitting to standing)	Pain intensity, neck disability, imagery influence, quality of life level.	VAS, NDI, MIQ-3, SF-36.	Pre- and post-intervention	4 weeks	No significant between-group differences found; MI provided no additional contribution. Pain and disability significantly decreased in both groups post-intervention. MI ability (kinesthetic and visual imagery) increased in both groups.
Suso-Martí, 2019 [23]	RCT	30 subjects (18–65 years) with chronic neck pain of at least 6 months	30. AOG: 10 MIG: 10 CG: 10	30.6 AOG: 33.5 MIG: 30.6 CG: 27.7	Women: AOG: 50% MIG: 50% CG: 60% Men: AOG: 50% MIG: 50% CG: 40%	MI group imagined two craniocervical flexion exercises with auditory cues (4 min total). AO group observed same exercises via video (4 min).	10 subjects viewed a video composed only of clips of nature landscapes for 4 min, without visualizing any motor gestures.	PPT in upper fibers of both trapezii, PPT in C2–C3 zygapophyseal joint, PPT in non-dominant lateral epicondyle, HR, motor imagery ability, mental chronometry, pain-related fear of movement, pain catastrophizing, neck disability, physical activity level, pain intensity.	Mechanical pressure algometer, Garmin Forerunner VR 225, MIQ-R, stopwatch, TAMPA, PCS, NDI, IPAQ, VAS.	One before the intervention, one immediately after, and one 10 min post-intervention. HR was measured at baseline, during, and immediately after the intervention.	1 day	Significant differences in cervical PPT were observed between the MI and AO groups, with AO producing greater modulation of local and remote pain and a stronger autonomic response. Both interventions induced immediate cervical pain modulation, which did not persist after 10 min. AO also elicited a higher HR response compared with MI.
León-Hernández, 2019 [24]	RCT	48 subjects (18–65 years) with chronic nonspecific neck pain (with at least 6 months of symptoms)	48. IG: 24 CG: 24	46.5 IG: 39.0 CG: 54.0	Women: IG 66.7% CG 83.3% Men: IG 33.33% CG 16.7%	24 subjects performed laterality discrimination task with neck images (20 images × 4 s)	24 subjects performed same task with object images.	Laterality discrimination task performance, cervical repositioning sense, active cervical ROM, pain-related fear of movement, pain catastrophizing, neck disability, pain intensity.	Recognize Online app, Motion Guidance Clinic Kit for JPE, CROM goniometer, TAMPA, PCS, NDI, VAS.	One before the intervention and one after the intervention.	1 day	Statistically significant changes in flexion, extension, and left rotation JPE found in the IG. Active cervical right rotation ROM significantly increased in IG vs. CG. No statistically significant differences found in CG.
Morales, 2020 [25]	RCT	54 asymptomatic subjects (18–65 years)	54. AO group: 19 MI group: 18 CG: 17	27.4 AOG: 30.6 MIG: 27.7 CG: 24.0	Women: AOG 6% MIG 41% CG 13% Men: AOG 94% MIG 59% CG 77%	AO group (*n* = 19) viewed cervical exercises dynamically; MI group (*n* = 18) performed implicit MI via laterality judgments.	CG (*n* = 17) performed cervical exercises (20 min, 3 × 12 reps).	Conditioned pain modulation, PPT in trapezius, PPT in tibialis anterior, active cervical ROM, neck flexor muscle endurance, ability to generate mental motor imagery, attention and cognitive function, physical activity level, anxiety, depression, pain-related fear of movement, pain catastrophizing, fear-avoidance beliefs.	Algometer, occlusion cuff, CROM goniometer, isometric test, MIQ-R, modified Stroop test with Encephalapp, IPAQ, STAI, BDI-II, TAMPA, PCS, FABQ.	One before the intervention and one after the intervention.	Each intervention lasted 5 days over a 2-week period.	Within-group changes observed in CG and AOG for conditioned pain modulation, cervical muscle endurance, and attention. Trapezius PPT changes observed in all three groups. ROM changes (flexion, extension, rotation) found in CG. AO improved motor imagery ability.
Suso-Martí, 2020 [26]	RCT	48 asymptomatic subjects (18–65 years)	48. IG: 24 CG: 24	32.5. IG: 34.8 CG: 30.1	Women: IG 62.5 CG 45.8 Men: IG 37.5 CG 54.2	24 subjects formed the intensive training group. OA + MI (2 × 20 reps, then 3 × 20 reps with orofacial tasks).	24 subjects formed the moderate training group that performed the same exercises as the intensive training group, but 10 repetitions.	PPT in masseter, PPT in temporalis, tongue muscle strength, perceived exertion, motor imagery ability, physical activity level.	Algometer, IOPI, Borg Scale, MIQ-R, IPAQ.	Four measurements: one before each intervention and one after each intervention.	2 days	Significant between-group differences in PPT; CG showed higher PPT than IG after session 2. Significant within-group differences in tongue strength observed only in CG (pre- vs. post-session 1).
Al Shrbaji, 2023 [27]	RCT	60 subjects (18–65 years) with chronic idiopathic neck pain of at least 3 months	60. IG: 30 CG: 30	39.9 IG: 37.4 CG: 42.3	Women: IG 80% CG 73.3% Men: IG 20% CG 26.7%	AOG: 11 min video showing two craniocervical flexion/extension exercises; included 4 min rest (black screen).	CG watched an 11 min video showing natural scenes without human motion stimuli.	Pain intensity, duration, and frequency, neck disability, pain catastrophizing, fear of movement, fear-avoidance beliefs, tactile acuity, neck muscle strength, trapezius PPT, PPT in C1–C2 and C5–C6 articular pillars.	VAS, NDI, PCS, TAMPA, FABQ, aesthesiometer (Baseline Two Point Aesthesiometer), hand dynamometer (Advanced Force Gauge), electronic pressure algometer.	One before the intervention and one after the intervention.	1 day	Similar acute hypoalgesic benefit obtained from single AO session and natural landscape observation (CG). No impact on kinesiophobia, fear-avoidance beliefs, or PPTs. AO had no positive effect on two-point discrimination or muscle strength.
La Touche, 2020 [28]	RCT	52 asymptomatic subjects (18–65 years)	52. CG: 13 VFG: 13 AOG: 13 MIG: 13	30.4 CG: 35.4 VFG: 32.5 AOG: 27.5 MIG: 26.2	Women: CG: 69.2% VFG: 46.2% AOG: 46.2% MIG: 46.2% Men: CG: 30.8% VFG: 53.8% AOG: 53.8% MIG: 53.8%	VFG (*n* = 13): Performed the control protocol while facing a mirror, focusing attention on the exercised region. MIG (*n* = 13): Viewed a craniomandibular video prior to the control protocol; instructed to generate kinesthetic imagery during execution. AOG (*n* = 13): Simultaneously observed a video while performing the control protocol actively and dynamically (external focus).	13 subjects performed a 20–25 min program of 8 temporomandibular, tongue, and facial exercises (8–10 repetitions, 30–60 seg rest)	Masseter PPT, temporalis PPT, tongue strength, maximum mouth opening, tongue length, ability to generate mental motor imagery, imagined gesture chronometry, ability to practice regular physical activity, physical activity level.	Mechanical pressure algometer, IOPI, cranio-mandibular scale, ruler and depressor, MIQ-R, stopwatch, Physical Activity Self-Efficacy Scale, IPAQ.	Four measurements in total: one pre-intervention and one after each intervention.	Three 45 min sessions on three consecutive days.	AO and MI with exercise may induce changes in masseter PPT. Only AO induced changes in tongue strength. AO, MI, and control groups improved in the MIQ-R visual subscale, while only AO improved in chronometry.
Cuenca-Martínez, 2019 [29]	RCT	30 subjects (18–65 years) with chronic nonspecific neck pain of at least 6 months	30. AOG: 10 MIG: 10 CG: 10	30.6 AOG: 33.5 MIG: 30.6 CG: 27.7	Women: AOG: 50% MIG: 50% CG: 60% Men: AOG: 50% MIG: 50% CG: 40%	MI group imagined two craniocervical flexion exercises with auditory cues (4 min total). AO group observed same exercises via video (4 min).	10 subjects viewed a video composed only of clips of nature landscapes for 4 min, without visualizing any motor gestures.	Cervical repositioning sense, ability to generate mental motor imagery, mental chronometry, pain-related fear of movement, neck disability, physical activity level.	Sensory Motor Control Oriented Rehabilitation IAOM-US, MIQ-R, stopwatch, TAMPA, NDI, IPAQ.	Three measurements in total: one before the intervention, one immediately after, and one 10 min post-intervention.	1 day	AO showed greater improvements vs. CG in cervical repositioning sense (extension: immediate & 10 min; flexion: 10 min). AO > MI in repositioning sense (extension: post-intervention). Both MI and AO groups outperformed CG in left rotation.

Abbreviations glossary (Table 1): RCT (randomized controlled trial), MI (motor imagery), AO (action observation), VR (virtual reality), IG (Intervention Group), CG (Control Group), AOG (Action Observation Group), MIG (Motor Imagery Group), VFG (Visual Feedback Group), VAS (Visual Analogue Scale), NDI (Neck Disability Index), MIQ-3 (Movement Imagery Questionnaire-3), SF-36 (Short Form-36 Health Survey), PPT (Pressure Pain Threshold), HR (Heart Rate), MIQ-R (Revised Movement Imagery Questionnaire), PCS (Pain Catastrophizing Scale), TAMPA (Tampa Scale for Kinesiophobia), IPAQ (International Physical Activity Questionnaire), ROM (Range of Motion), CROM (Cervical Range of Motion), JPE (Joint Position Error), STAI (State-Trait Anxiety Inventory), BDI-II (Beck Depression Inventory-II), FABQ (Fear-Avoidance Beliefs Questionnaire), IOPI (Iowa Oral Performance Instrument), IAOM (International Academy of Orthopedic Medicine).

**Table 2:** Methodological score of RCTs using the PEDro scale.

Study	1	2	3	4	5	6	7	8	9	10	11	Total
Özcan et al., 2018 [22]	Y	Y	Y	Y	N	N	Y	Y	Y	Y	Y	8
Suso-Martí et al., 2019 [23]	Y	Y	Y	Y	N	N	Y	Y	Y	Y	Y	8
León-Hernández et al., 2019 [24]	Y	Y	Y	Y	N	N	Y	Y	Y	Y	Y	8
Morales et al., 2020 [25]	Y	Y	Y	Y	N	Y	Y	Y	Y	Y	Y	9
Suso-Martí et al., 2020 [26]	Y	Y	Y	Y	N	N	Y	Y	Y	Y	Y	8
Al Shrbaji et al., 2023 [27]	Y	Y	Y	Y	N	N	N	Y	Y	Y	Y	8
La Touche et al., 2020 [28]	Y	Y	Y	Y	N	Y	Y	Y	Y	Y	Y	9
Cuenca-Martínez et al., 2019 [29]	Y	Y	Y	Y	N	N	Y	Y	Y	Y	Y	8

Y—Yes; N—No.

## Data Availability

The data supporting the findings of this study are available from the corresponding author upon reasonable request due to privacy, ethical approvals, and data protection regulations of the primary studies.

## Supplementary Materials

The following supporting information can be downloaded at: https://www.mdpi.com/article/10.3390/life16010145/s1, Data S1: The retrieval strategy.

## Abbreviations

The following abbreviations are used in this manuscript:

MRTs	Movement Representation Techniques
MI	Motor imagery
AO	Action observation
TMDs	Temporomandibular disorders
RCTs	Randomized clinical trials
PROSPERO	International Prospective Register of Systematic Reviews
PRISMA	Preferred Reporting Items for Systematic Reviews and Meta-Analyses
PEDro	Physiotherapy Evidence Database
RoB 2	Cochrane Risk of Bias tool for randomized trials (version 2)
PPT	Pressure Pain Threshold
VFT	Visual feedback therapy
TMJ	Temporomandibular joint
PICOS	Population, Intervention, Comparison, Outcomes, and Study design
WOS	Web of Science
AGA	Alberto García-Alonso
RMS	Roberto Méndez-Sánchez
LPF	Luis Polo-Ferrero
IG	Intervention group
CG	Control group
VAS	Visual Analogue Scale
ROM	Range of Motion
IOPI	Iowa Oral Performance Instrument
MIQ-R	Revised Movement Imagery Questionnaire
MIQ-3	Movement Imagery Questionnaire-3
FABQ	Fear Avoidance Beliefs Questionnaire
STAI	The State-Trait Anxiety Inventory
BDI-II	Beck Depression Inventory-II
IPAQ	International Physical Activity Questionnaire
SF-36	Short Form 36 Health Survey
HR	Heart rate
