# Supplementary material for: Neurorehabilitation-Based Movement Representation Techniques in the Management of Craniocervical and Orofacial Pain: A Systematic Review of Randomized Controlled Trials
Source: Life (Basel). 2026 Jan 15;16(1):145. doi: 10.3390/life16010145 (PMC12842662; doi:10.3390/life16010145)
Supplement: Supplementary file 1 [file life-16-00145-s001.zip › life-4039458-supplementary.pdf]

Supplementary Data S1. The retrieval strategy.

PubMed Search Formula: 91

("motor imagery"[All Fields] OR "graded motor imagery"[All Fields] OR "GMI"[All Fields] OR "mirror therapy"[All Fields] OR "visual mirror feedback"[All Fields] OR "neuroscience education"[All Fields]) AND ("cervical"[All Fields] OR "neck pain"[All Fields] OR "craniocervical"[All Fields] OR "craniomandibular"[All Fields] OR "temporomandibular disorders"[All Fields] OR "temporomandibular joint"[All Fields] OR "orofacial"[All Fields] OR "face and head pain"[All Fields])

Scopus Search Formula: 115

TITLE-ABS-KEY (("motor imagery" OR "graded motor imagery" OR "GMI" OR "mirror therapy" OR "visual mirror feedback" OR "neuroscience education") AND ("cervical" OR "neck pain" OR "craniocervical" OR "craniomandibular" OR "temporomandibular disorders" OR "temporomandibular joint" OR "orofacial" OR "face and head pain"))

Web of Science (WOS) Search Formula: 174

("motor imagery" OR "graded motor imagery" OR "GMI" OR "mirror therapy" OR "visual mirror feedback" OR "neuroscience education") AND ("cervical" OR "neck pain" OR "craniocervical" OR "craniomandibular" OR "temporomandibular disorders" OR "temporomandibular joint" OR "orofacial" OR "face and head pain") (Topic)

EBSCO Host Search Formula: 117

("motor imagery" OR "graded motor imagery" OR "GMI" OR "mirror therapy" OR "visual mirror feedback" OR "neuroscience education") AND ("cervical" OR "neck pain" OR "craniocervical" OR "craniomandibular" OR "temporomandibular disorders" OR "temporomandibular joint" OR "orofacial" OR "face and head pain")
